# Effect of Atheromatous Aorta on Thromboembolic Complications after Endovascular Aortic Aneurysm

**DOI:** 10.3400/avd.oa.20-00072

**Published:** 2020-09-25

**Authors:** Tsunehiro Shintani, Hiroshi Mitsuoka, Yuto Hasegawa, Masanori Hayashi, Kayoko Natsume, Kazuhiro Ookura, Yasunori Sato, Hideaki Obara

**Affiliations:** 1 Department of Vascular Surgery, Shizuoka Red Cross Hospital; 2 Department of Cardiovascular Surgery, Shizuoka Hospital; 3 Department of Cardiac Surgery, Shizuoka Red Cross Hospital; 4 Department of Preventive Medicine and Public Health, Keio University School of Medicine; 5 Department of Surgery, Keio University School of Medicine

**Keywords:** atheromatous aorta, endovascular aortic aneurysm repair, medication

## Abstract

**Objective**: The purpose of this study was to evaluate the effect of atheromatous aorta on thromboembolic complications after endovascular aortic aneurysm repair (EVAR) and to assess the risk factors for these complications.

**Materials and Methods**: This retrospective study included patients who underwent EVAR for an abdominal aortic aneurysm at the Shizuoka Red Cross Hospital from 2007 to 2018. We defined atheromatous aorta as a thoracic shaggy aorta or abdominal aorta with neck thrombus. The main outcome was renal dysfunction and peripheral embolization (thromboembolic complications). We compared the incidence of thromboembolic complications between patients with normal aorta and atheromatous aorta. Moreover, we assessed the risk factors associated with thromboembolic complications in patients with atheromatous aorta.

**Results**: Patients with atheromatous aorta had significantly more thromboembolic complications, such as renal dysfunction (24.5% vs. 3.9%; P<0.001) and peripheral embolization (12.3% vs. 0.0%; P<0.001) than those with normal aorta, respectively. We identified no risk factors associated with thromboembolic complications in patients with atheromatous aorta.

**Conclusion**: Atheromatous aorta increases the risk of thromboembolic complications after EVAR. However, there is no established therapy for these thromboembolic complications. Further studies are necessary to determine the appropriate therapy, including appropriate preoperative medication, to prevent these complications.

## Introduction

Endovascular aortic aneurysm repair (EVAR) for abdominal aortic aneurysm (AAA) has become a standard treatment for high-risk surgical patients with a suitable anatomy.^1)^ Extensive atheromatous aorta is frequently associated with AAA, and the indication of EVAR for AAA in patients with atheromatous aorta is a controversial issue.^2–5)^ A thoracic shaggy aorta refers to an atheromatous aorta with an irregular and spiculated shape from the aortic arch to the visceral segment.^2,3)^ A thoracic shaggy aorta is considered a relative contraindication to EVAR because manipulation of catheters can sometimes lead to catastrophic embolization.^6)^ An abdominal aorta with neck thrombus also carries a high risk of thromboembolism because the aneurysmal neck is commonly the attachment site of a proximal stent-graft, and neck thrombus can be released from the neck and cause embolization.^3,7)^ A previous report recommended open surgery for AAA with an extensive atheromatous aorta^3)^; however, patients’ conditions sometimes preclude open surgery. Therefore, the results of EVAR for AAA in patients with atheromatous aorta need to be clarified.

Supportive therapy, including correcting risk factors, taking statins and antiplatelet drugs, and cessation of intake of anticoagulation, is advocated to reduce thromboembolic complications in aortic surgery.^8–11)^ However, there is no established evidence showing the usefulness of preoperative medication to prevent these thromboembolic complications in EVAR. Therefore, the purpose of this study was to evaluate the effect of atheromatous aorta on thromboembolic complications after EVAR and to assess the risk factors, including preoperative medications, for these complications.

## Patients and Methods

### Patients and study setting

This retrospective study included all patients who underwent EVAR from 2007 to December 2018 in the Shizuoka Red Cross Hospital. Data collection was performed until December 2019. In this study, the following four types of commercially available stent grafts were used: Excluder (W. L. Gore and Assoc., Flagstaff, AZ, USA), Zenith or Zenith Flex (Cook Inc., Bloomington, IN, USA), Endurant (Medtronic, Santa Rosa, CA, USA), and AFX (Endologix, Irvine, CA, USA). Data were collected in a prospectively accumulated database. Inflammatory, infectious, or ruptured AAAs were excluded. Data from patients who had no preoperative enhanced computed tomography (CT) images of the thoracic aorta were also excluded. Patients who lost the renal artery during EVAR and those with hydronephrosis owing to the aneurysm were also excluded.

### EVAR procedure and postoperative follow-up

EVAR was performed under the instruction of two board-certified vascular surgeons (TS and HM) who were approved by the Japanese Committee for Stent-graft Management. When EVAR was performed in patients with atheromatous aorta, we avoided the atheromatous neck as the proximal landing zone and withheld proximal ballooning under gentle catheter manipulation. Although device selection was according to the physician’s preference, we refrained from using a suprarenal fixation device (Zenith, Zenith Flex, Endurant, or AFX with suprarenal proximal extension) in the presence of a suprarenal thrombus.^7)^

Preoperative enhanced CT imaging with a slice thickness of ≤2.5 mm was performed for EVAR planning. Preoperative serum creatinine and low-density lipoprotein cholesterol (LDL-C) levels were measured just before EVAR. All patients underwent regular follow-up evaluations and measurement of serum creatinine levels 1, 6, and 12 months after EVAR, and yearly thereafter. This study was approved by the institutional review board of our hospital.

### Data collection and outcomes

Data were collected for patients’ demographics, comorbidities, aortic pathology, type of stent-graft device, and preoperative medication. Chronic kidney disease (CKD) was defined as an estimated glomerular filtration rate of <60 mL/min/1.73 m^2^, which was calculated from serum creatinine concentrations.

The definition of an atheromatous aorta was a thoracic shaggy aorta (shaggy aorta) or neck thrombus. A shaggy aorta was defined as a diffuse atheromatous plaque involving >75% of the length of the thoracic aorta from the aortic arch to the visceral segment with a thickness of >4 mm.^2)^ The definition of a neck thrombus was an infrarenal thrombus located in the first 10 mm of the aortic neck with a thickness of >2 mm and constituting >25% of the neck circumference, or a suprarenal thrombus associated with a suprarenal fixation device.^7)^ The presence of an atheromatous aorta in preoperative CT images was verified by three vascular surgeons (TS, MH, and KN).

Stent-graft devices were divided into the two following groups: infrarenal fixation devices (Excluder and AFX with infrarenal proximal extension) and suprarenal fixation devices.

Preoperative medication included antiplatelet drugs, anticoagulants, statins, angiotensin-converting enzyme inhibitors or angiotensin receptor blockers, and β-blockers, which were prescribed at least 30 days before EVAR.

The main outcome was renal dysfunction and peripheral embolization (thromboembolic complications). Renal dysfunction was defined as deterioration of the glomerular filtration rate category (that is, CKD “G” stage) 6 months after EVAR. Some patients’ data regarding renal dysfunction 6 months after EVAR were missing. Patients receiving maintenance hemodialysis before EVAR were excluded from the evaluation of renal dysfunction. Peripheral embolization was defined as lower extremity artery embolism, including blue toe syndrome, within 6 months after EVAR.

### Risk factors associated with thromboembolic complications in patients with atheromatous aorta

We assessed the risk factors associated with thromboembolic complications in patients with atheromatous aorta and considered age, sex, comorbidities, suprarenal fixation device, preoperative medication profile, and serum LDL-C levels as risk factors for thromboembolic complications.

### Statistical analysis

Continuous variables were presented as mean±standard deviation and were compared with t-test or Mann–Whitney U test. Categorical variables were presented as frequencies and percentages and were compared with the chi-squared test or Fisher’s exact test.

The overall survival rates between patients with normal aorta and those with atheromatous aorta were assessed by the Kaplan–Meier method with the log-rank test. Univariate analyses of variables affecting the risk of renal dysfunction or peripheral embolization in patients with atheromatous aorta were performed to express the odds ratios and 95% confidence intervals. Variables with P<0.10 in the univariate analysis were incorporated into the logistic regression analysis for the multivariate analysis.

All P-values were two-sided, and P<0.05 indicated a statistically significant difference. All analyses were performed using IBM SPSS version 24 (IBM Corp., Armonk, NY, USA).

## Results

### Patient population

**Figure 1:**
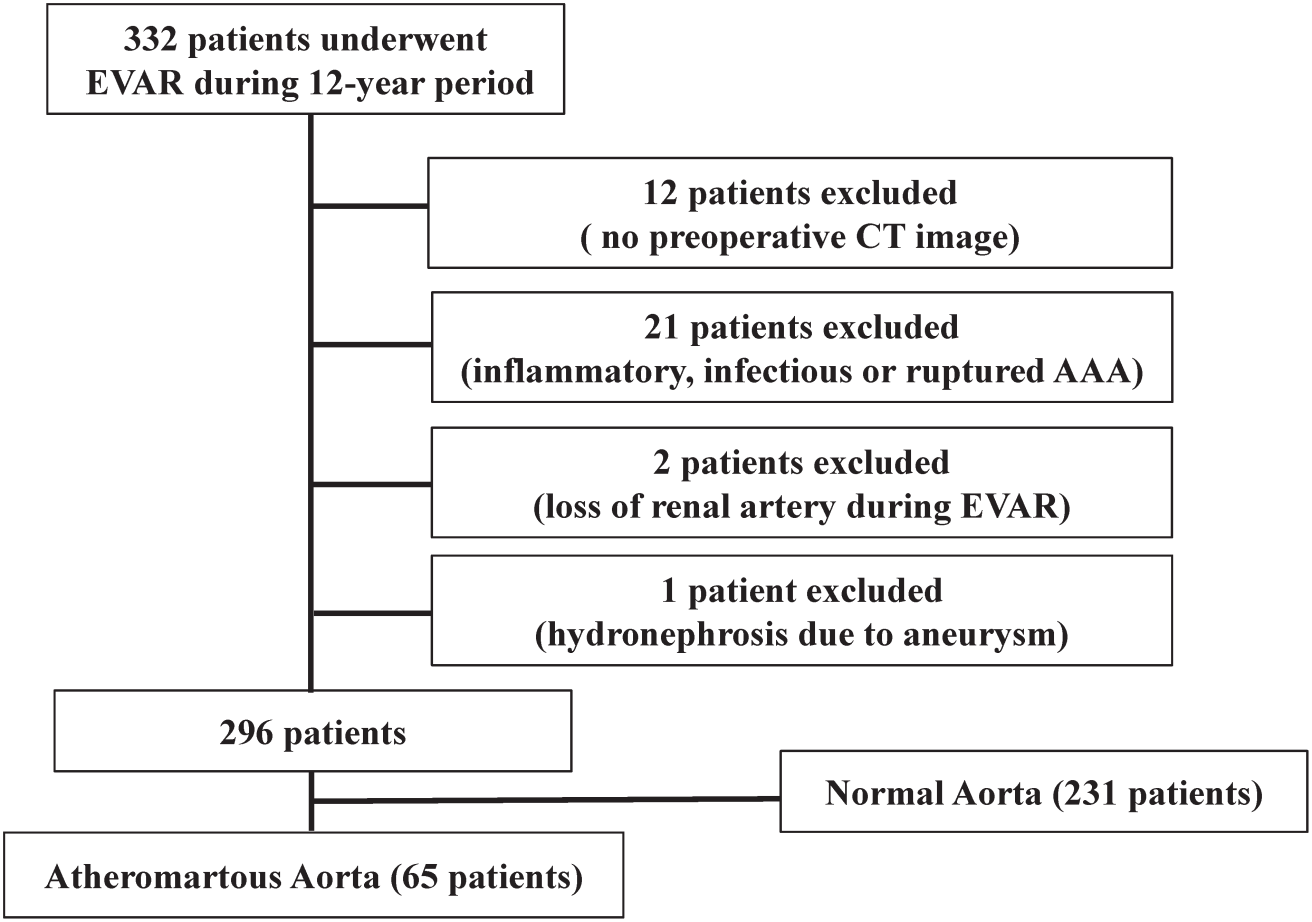
Fig. 1 Flow chart of patients in this study.

Three hundred and thirty-two consecutive patients underwent EVAR from 2007 to 2018. We excluded 36 patients who had no preoperative enhanced thoracic CT images (n=12); inflammatory, infectious, or ruptured AAA (n=21); loss of the renal artery during EVAR (n=2); and hydronephrosis owing to the aneurysm (n=1). The remaining 296 patients were eligible for inclusion in the study. This population was divided into the following two groups: normal aorta (231 patients) and atheromatous aorta (65 patients; **Fig. 1**).

Patients’ demographics, comorbidities, aortic pathology, type of stent-graft device, medication, and LDL-C levels in each group are shown in **Table 1**. The proportions of male sex, diabetes mellitus, coronary artery disease, cerebrovascular disease, hemodialysis, and history of smoking were higher in patients with an atheromatous aorta than in those with normal aorta. Antiplatelets were used more frequently in patients with an atheromatous aorta than in those with normal aorta. There was no significant difference in the type of stent-graft or the use of a suprarenal fixation device between patients with normal aorta versus an atheromatous aorta.

**Table 1:** Table 1 Baseline demographic data, comorbidities, aortic pathology, stent-graft device, pharmacotherapy, and LDL-C levels in all patients and in those with atheromatous aorta

Variable	All patients			Atheromatous aorta		
	Normal aorta (n=231)	Atheromatous aorta (n=65)	P	Shaggy aorta (n=20)	Neck thrombus only (n=45)	P
Demographic data						
Age, years	75.8±8.2	74.8±6.4	0.39	74.2±4.9	75.0±7.0	0.55
Male sex	191 (82.7)	61 (93.8)	0.025	19 (95.0)	42 (93.3)	1.00
Follow-up, months	46.3±34.5	49.5±33.3	0.53	47.5±35.7	50.4±32.6	0.75
Comorbidities						
Hypertension	164 (71.0)	52 (80.0)	0.15	18 (90.0)	34 (75.6)	0.31
Diabetes mellitus	20 (8.7)	12 (18.5)	0.025	4 (20.0)	8 (17.8)	1.00
Coronary artery disease	62 (26.8)	29 (44.6)	0.006	7 (35.0)	22 (48.9)	0.30
Cerebrovascular disease	35 (15.2)	24 (36.9)	<0.001	7 (35.0)	17 (37.8)	0.83
COPD	40/213 (18.8)	15/60 (25.0)	0.29	7 (35.0)	8/40 (20.0)	0.21
CKD (eGFR <60 mL/min/1.73m^2^)	119 (51.5)	40 (61.5)	0.15	15 (75.0)	25 (55.6)	0.14
Hemodialysis	5 (2.2)	5 (7.7)	0.03	4 (20.0)	1 (2.2)	0.028
Smoking history	135 (59.7)	54 (83.1)	<0.001	18 (90.0)	36 (80.0)	0.48
Aortic pathology						
Shaggy aorta	0 (0.0)	20 (30.8)	NA	NA	NA	NA
Neck thrombus	0 (0.0)	59 (92.3)	NA	NA	NA	NA
Stent-graft device						
Suprarenal fixation	115 (49.8)	34 (52.3)	0.72	8 (40.0)	26 (57.8)	0.19
Excluder	112 (48.5)	30 (46.2)		11 (55.0)	19 (42.2)	
Zenith or Zenith Flex	71 (30.7)	21 (32.3)		4 (20.0)	17 (37.8)	
Endurant	38 (16.5)	12 (18.5)	0.98	3 (15.0)	9 (20.0)	0.14
AFX (infrarenal fixation)	4 (1.7)	1 (1.5)		1 (5.0)	0 (0.0)	
AFX (suprarenal fixation)	6 (2.6)	1 (1.5)		1 (5.0)	0 (0.0)	
Pharmacotherapy						
Antiplatelet	83 (35.9)	38 (58.5)	0.01	11 (55.0)	27 (60.0)	0.71
Anticoagulation	23 (10.0)	10 (15.4)	0.22	5 (25.0)	5 (11.1)	0.26
Statin	83 (35.9)	27 (41.5)	0.41	10 (50.0)	17 (37.8)	0.36
ACEI/ARB	120 (51.9)	37 (56.9)	0.48	11 (55.0)	26 (57.8)	0.84
ß-blocker	38 (16.5)	15 (23.1)	0.22	6 (30.0)	9 (20.0)	0.52
LDL-C (mg/dL)	111.3±31.2	118.3±33.7	0.28	114.5±36.7 (19)	119.7±32.5 (41)	0.59
≤100 mg/dL	123/199 (61.8)	41/60 (68.3)	0.36	13/19 (68.4)	28/41 (68.3)	0.99

Data are presented as mean±standard deviation for continuous variables and n (%) for categorical variables unless otherwise indicated. Some patients’ data regarding COPD and LDL-C levels were missing. COPD: chronic obstructive pulmonary disease; CKD: chronic kidney disease; eGFR: estimated glomerular filtration rate; ACEI: angiotensin-converting enzyme inhibitor; ARB: angiotensin receptor blocker; LDL-C: low-density lipoprotein cholesterol; NA: not applicable

### Outcome in all patients

**Figure 2:**
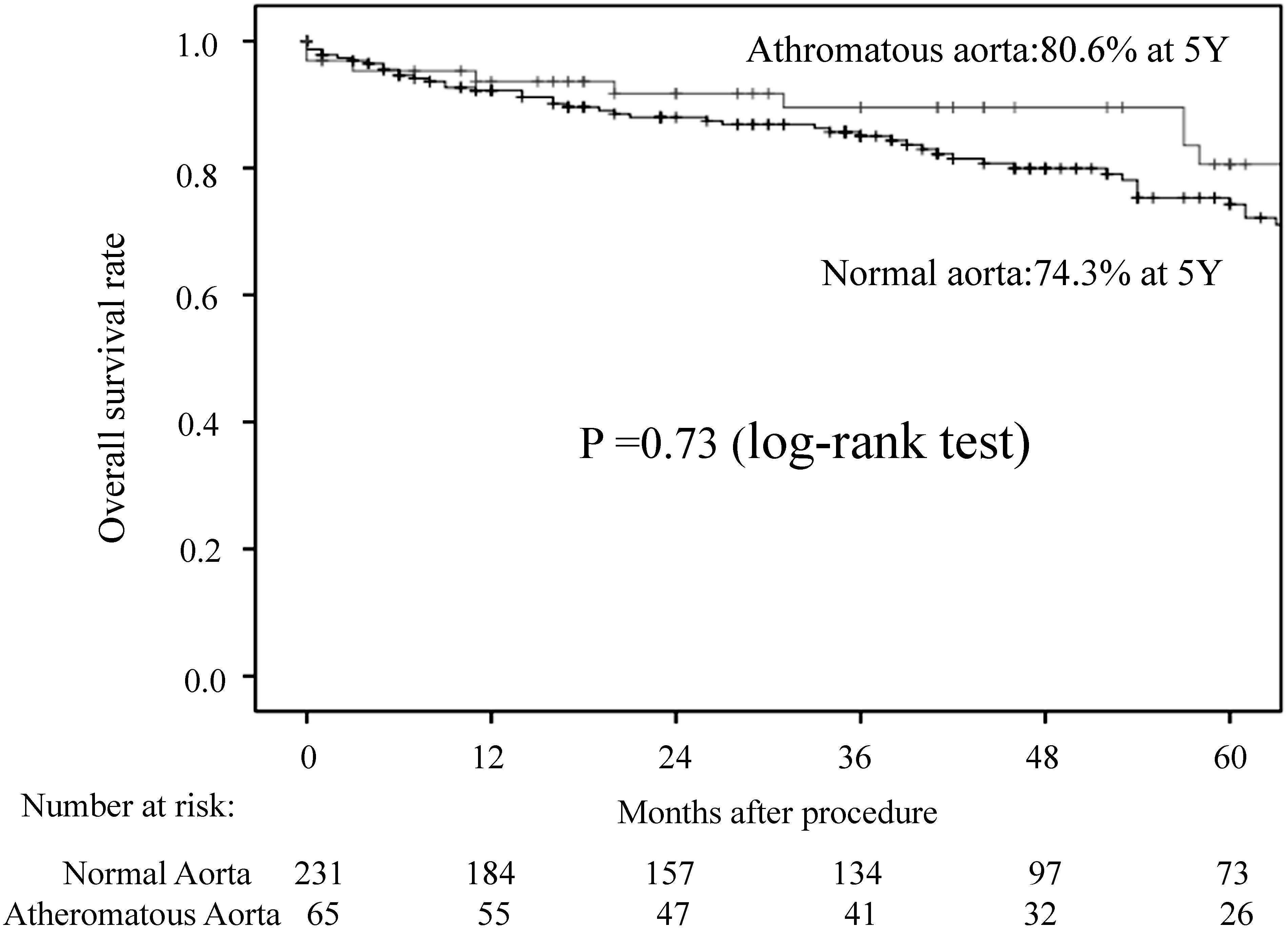
Fig. 2 Kaplan–Meier curves showing the 5-year overall survival rates in patients with normal aorta and in those with atheromatous aorta.

We experienced 13 cases of renal dysfunction and 8 of peripheral embolization in patients with atheromatous aorta. Patients with atheromatous aorta had significantly more thromboembolic complications, such as renal dysfunction (13/53: 24.5% vs. 7/181: 3.9%; P<0.001) and peripheral embolization (8/65: 12.3% vs. 0/231: 0.0%; P<0.001), than those with normal aorta. The details of peripheral embolization are shown in **Table 2**. All cases of peripheral embolization occurred in patients with atheromatous aortas. Two cases of fatal massive embolism with multiple organ failure occurred with peripheral embolization, and both patients died immediately after EVAR. The CKD stage in four patients deteriorated, and three patients required permanent hemodialysis. Three cases of blue toe syndrome were late onset (Cases 6, 7, and 8). The 5-year overall survival rates were not significantly different between patients with normal aorta and atheromatous aorta (**Fig. 2**).

**Table 2:** Table 2 Details of peripheral embolization in patients with atheromatous aorta

Case	Aortic pathology	Event	Time	Renal dysfunction	Outcome
1	Neck thrombus	Massive embolism	POD 0	NA	Death due to massive embolism at POD 3
2	Shaggy Ao/neck thrombus	Blue toe syndrome	POD 7	Originally HD	Death due to pancreatic cancer at POM 4
3	Neck thrombus	SFA embolism	POD 0	None	Alive at POM 21
4	Shaggy Ao/neck thrombus	Massive embolism	POD 0	Originally HD	Death due to massive embolism at POD 1
5	Neck thrombus	Blue toe syndrome	POD 0	Deterioration	Alive at POM 18, permanent HD
6	Shaggy Ao	Blue toe syndrome	POM 3	Deterioration	Death due to pneumonia at POM 31
7	Shaggy Ao/neck thrombus	Blue toe syndrome	POM 6	Deterioration	Alive at POM 10, permanent HD
8	Shaggy Ao/neck thrombus	Blue toe syndrome	POM 1	Deterioration	Alive at POM 10, permanent HD

Time: time to event; renal dysfunction: renal dysfunction at 6 months after EVAR; shaggy Ao: shaggy aorta; SFA: superficial femoral artery; POD: postoperative day; POM: postoperative month; NA: not applicable; HD: hemodialysis

### Patient population with atheromatous aorta

There were 20 patients with a shaggy aorta and 45 with neck thrombus (without a shaggy aorta) among those with atheromatous aorta (**Table 1**). A higher proportion of patients with a shaggy aorta had undergone hemodialysis compared with those with neck thrombus. We found no significant difference regarding preoperative medications, type of stent-graft, and use of a suprarenal fixation device between patients with a shaggy aorta and those with neck thrombus.

### Outcomes in patients with atheromatous aorta

There were no significant differences in the rate of thromboembolic complications (renal dysfunction [6/16: 37.5% vs. 7/37: 18.9%, respectively; P=0.18] and peripheral embolization [5/20: 2.5% vs. 3/45: 6.7%, respectively; P=0.10]) between patients with a shaggy aorta and those with neck thrombus.

### Risk factor analysis for thromboembolic complications in patients with atheromatous aorta

There were no statistically significant risk factors in the univariate analysis of variables that affected the risk of renal dysfunction in patients with atheromatous aorta (**Table 3**). Coronary artery disease, hemodialysis, and anticoagulation were possible risk factors in the univariate analysis of variables that affected the risk of peripheral embolization. However, there were no statistically significant risk factors in the multivariable analysis (**Table 3**).

**Table 3:** Table 3 Univariate and multivariate analysis of variables affecting the risk of renal dysfunction or peripheral embolization in patients with atheromatous aorta

Variable	Renal dysfunction		Peripheral embolization			
	Univariate analysis		Univariate analysis		Multivariate analysis	
	OR (95%CI)	P	OR (95%CI)	P	OR (95%CI)	P
Age						
≥80 vs. <80 years	1.43 (0.33–6.13)	0.63	0.54 (0.12–2.57)	0.44	NA	NA
Sex						
male vs. female	7.09 (0.59–85.7)	0.12	2.57 (0.23–28.2)	0.44	NA	NA
Comorbidities						
Hypertension	1.20 (0.27–5.41)	0.81	0.54 (0.06–4.79)	0.58	NA	NA
Diabetes mellitus	1.17 (0.21–6.47)	0.86	0.31 (0.06–1.55)	0.15	NA	NA
Coronary artery disease	0.70 (0.20–2.46)	0.58	6.76 (0.78–58.5)	0.08	8.08 (0.85–76.9)	0.07
Cerebrovascular disease	1.18 (0.33–4.26)	0.80	0.54 (0.12–2.40)	0.42	NA	NA
COPD	0.39 (0.09–1.71)	0.21	0.63 (0.10–3.87)	0.62	NA	NA
CKD (eGFR <60 mL/min/1.73m^2^)	0.94 (0.26–3.39)	0.92	0.96 (0.21–4.40)	0.95	NA	NA
Hemodialysis	NA	NA	0.17 (0.02–1.21)	0.08	0.15 (0.01–1.57)	0.11
Smoking history	0.86 (0.16–4.75)	0.86	1.78 (0.31–10.2)	0.52	NA	NA
Stent-graft device						
Suprarenal fixation	3.04 (0.80–11.6)	0.09	1.61 (0.35–7.38)	0.54	NA	NA
Medical treatment						
Antiplatelet therapy	1.43 (0.40–5.06)	0.58	1.47 (0.34–6.52)	0.61	NA	NA
Anticoagulation	0.37 (0.07–1.93)	0.24	0.23 (0.05–1.20)	0.08	0.18 (0.03–1.19)	0.08
Statins	0.42 (0.12–1.51)	0.18	2.34 (0.44–12.6)	0.32	NA	NA
ACEI/ARB	1.94 (0.55–6.89)	0.30	1.38 (0.31–6.05)	0.67	NA	NA
ß-blocker	0.34 (0.09–1.35)	0.13	0.89 (0.16–4.93)	0.89	NA	NA
LDL-C						
≤100 vs. >100 mg/dL	1.08 (0.28–4.31)	0.91	0.32 (0.04–2.90)	0.31	NA	NA

OR: odds ratio; CI: confidence interval; COPD: chronic obstructive pulmonary disease; CKD: chronic kidney disease; eGFR: estimated glomerular filtration rate; ACEI: angiotensin-converting enzyme inhibitor; ARB: angiotensin receptor blocker; LDL-C: low-density lipoprotein cholesterol

## Discussion

Previous studies showed that a shaggy aorta was associated with high morbidity and mortality secondary to catastrophic embolization after EVAR.^2,4,5)^ Additionally, aortic neck thrombus caused embolic complications related to cholesterol embolization after EVAR.^3,7)^ Both of these conditions need to be considered together to better understand the occurrence of thromboembolic complications in EVAR with atheromatous aorta. However, there are few studies including both conditions.^3)^ We defined atheromatous aorta as a thoracic shaggy aorta or neck thrombus and included both conditions in this study. In the current study, the background data of both conditions were similar, except for the number of patients receiving hemodialysis. There were no significant differences in thromboembolic complications after EVAR between both conditions.

Most of the thromboembolic complications after EVAR are related to cholesterol embolization syndrome (CES).^3,7)^ CES appears when atheromatous aortic plaques rupture, spreading atheromatous debris into small- or medium-caliber arteries. This causes end-organ damage by mechanical obstruction and an inflammatory response.^9–11)^ The special feature of CES is a multitude of small emboli that occur over time. In this study, three cases of blue toe syndrome developed as late complications in patients with atheromatous aorta. We noticed this complication only after patients complained of painful ulcer and gangrene in the lower extremity digits in our outpatient clinic. We believe that blue toe syndrome worsened insidiously over time in these patients. A previous study discussed a case of blue toe syndrome in an EVAR patient with a massive neck atheroma as a late complication and reported that the presentation of CES is sometimes delayed for weeks or months after the procedure.^3)^ There is a need to pay attention to thromboembolic complications for a long time after EVAR, when patients with atheromatous aorta undergo EVAR.

In our study, patients with atheromatous aorta had higher proportions of male sex, diabetes mellitus, coronary artery disease, cerebrovascular disease, hemodialysis, and a history of smoking. This result is similar to that in a previous study showing that the usual risk factors for atherosclerosis are also major risk factors for CES.^10)^

A wide variety of clinical presentations of CES have been reported. Among them, the kidney and skin are the most commonly affected organs.^10)^ The bowel is also an affected organ, but a diagnosis of CES in the bowel is relatively difficult because ischemic colitis is caused by a reduction in intestinal blood flow during EVAR, and a definite diagnosis requires invasive organ biopsy.^3)^ Therefore, we did not include ischemic colitis as a thromboembolic complication. We experienced seven cases of skin (peripheral) presentations (five cases of blue toe syndrome and two of massive embolism), but only in patients with atheromatous aorta. A diagnosis of CES in the kidney is sometimes difficult, but kidney impairment caused by CES appears subacutely and is clearly distinguished from contrast agent-related kidney impairment, which appears acutely.^3,7,12)^ We previously found that kidney function 6 months after EVAR reflected the effects of CES most accurately.^7)^ We then defined the kidney impairment in this phase as a thromboembolic complication. Consequently, we found that patients with atheromatous aorta had significantly more thromboembolic complications, such as renal dysfunction and peripheral embolization, compared with those with normal aorta, which is consistent with previous studies.^2–5,7)^

In our study, the 5-year overall survival rates were not significantly different between patients with normal aorta and those with an atheromatous aorta. This result is different from that of a previous study, which showed that patients with a shaggy aorta had a significantly higher mortality than those normal aorta.^2)^ However, the authors of the study suggested that the worse survival seen in patients with shaggy aorta was secondary to comorbidities, and atheromatous aorta may not directly affect long-term survival.

Endovascular techniques, such as avoiding the atheromatous neck as the proximal landing zone, withholding proximal ballooning, and gentle catheter manipulation, are desirable for preventing thromboembolic complications after EVAR in patients with atheromatous aorta. However, evaluating the usefulness of these techniques is difficult. A previous report suggested a risk of renal microembolization after EVAR when using a suprarenal fixation device.^13)^ In our study, using a suprarenal fixation device in patients with atheromatous aorta was not a risk factor for thromboembolic complications. This difference may be because of our policy of refraining from using a suprarenal fixation device in the presence of a suprarenal thrombus to reduce thromboembolic complications.^7)^ Although a successful case report of thoracic EVAR with massive aortic plaques using a temporary intra-aortic filter was reported, this is not a practical technique.^14)^ Currently, there are no valid EVAR techniques to reduce thromboembolic complications after EVAR. Therefore, supportive therapy, especially preoperative medication, is the mainstay for reducing thromboembolic complications after EVAR. Among the possible preoperative medications, using statins is the most attractive strategy. There is some evidence that statin therapy decreases the risk of CES.^9–11)^ This beneficial role of statins could be related to plaque stabilization and regression through lipid-lowering and anti-inflammatory mechanisms. Statins and a reduction in LDL-C levels are associated with regression of thoracic atherosclerotic lesions.^15–17)^ Another study showed that preoperative stains reduced the length of hospital stay in patients undergoing EVAR.^8)^ However, there is no direct evidence that statin therapy prevents CES after EVAR. In our study, univariate analysis showed that statins and a reduction in LDL-C levels were not associated with thromboembolic complications.

Use of other preoperative medications, including antiplatelet drugs or antihypertensive agents, appears reasonable because such agents prevent cardiovascular events, which are risk factors in patients with atheromatous aorta.^9–11)^ However, there is no direct evidence that these agents prevent CES after EVAR. Anticoagulation may lead to plaque rupture and subsequent CES, and cessation of anticoagulation therapy is recommended in patients with CES. However, the causal relationship between anticoagulation and CES is unclear. Multivariate analysis revealed that anticoagulation did not significantly affect the risk of peripheral embolization in this study.

Previous history of thromboembolic events may be a risk factor for thromboembolic complications after EVAR. However, we experienced only one such case (blue toe syndrome) before EVAR in this study. In the affected patient, mural thrombus in the AAA was considered to be the source of the embolism, and blue toe syndrome improved after EVAR. Evaluating more cases is necessary to determine the risk of thromboembolic complication during EVAR for patients with a history of thromboembolic events.

Our study has limitations. First, this was a retrospective study. Second, the decision regarding whether to perform EVAR or open surgery for AAA with atheromatous aorta and the stent-graft choice, which are considered selection biases, were dependent on the attending physician. Third, we limited the occurrence of thromboembolic complications to within 6 months after EVAR for the same reason as that in a previous study, that is, that the association between EVAR and complications becomes less clear beyond 6 months after EVAR.^3)^ However, we cannot exclude the possibility that late-onset thromboembolic complications may be secondary to spontaneous plaque rupture,^11)^ which is another limitation of our study. Finally, we found no association between preoperative medication and thromboembolic complications in patients with atheromatous aorta. However, we had no strict criteria regarding preoperative medications, especially regarding the dosage and usage period of statins or the target LDL-C level before EVAR. Further studies under a strict protocol are necessary to determine the role of preoperative medication in preventing these complications after EVAR.

## Conclusion

Atheromatous aorta increases the risk of thromboembolic complications after EVAR, and most of the thromboembolic complications after EVAR are related to CES. Although there is some evidence that supportive therapy using medication decreases the risk of CES, direct evidence for reducing thromboembolic complications after EVAR in patients with atheromatous aorta is insufficient. Further studies are necessary to determine the role of preoperative medication in preventing these complications.

## Additional Note

A summary of this study was presented at the 60th Annual Meeting of the Japanese College of Angiology on October 11, 2019 in Tokyo, and it received a recommendation by the chairperson.
